# Special Issue: Food Bioactive Peptides

**DOI:** 10.3390/ijms232415985

**Published:** 2022-12-15

**Authors:** Leticia Mora, Fidel Toldrá

**Affiliations:** Instituto de Agroquímica y Tecnología de Alimentos (CSIC), Avenue Agustín Escardino 7, 46980 Paterna, Spain

This Special Issue of the *International Journal of Molecular Sciences* is focused on bioactive peptides in foods or hydrolyzates of food by-products, the methods for the extraction and purification of bioactive peptides, their structural and functional characterization, and the mechanisms of action that regulate their activity and support the reported health benefits. It also contemplates those studies on the bioavailability of particular bioactive peptides using simulated gastrointestinal digestion and intestinal transport, together with the confirmation of their health benefits, as well as the recent advances and challenges in the use of proteomics and peptidomics for the analysis of bioactive peptides.

This Special Issue includes papers from nine research groups that have been invited to publish their recent advances and developments within this field. The first manuscript, authored by Martineau-Côté and co-workers [1], reported the potential health-beneficial activities of three Canadian faba bean varieties (Fabelle, Malik and Snowbird) after in vitro gastrointestinal digestion, and they were compared to two commonly used legumes (peas and soy). Eleven faba bean peptides and their predicted bioactivities were identified through in silico methodology. The second manuscript, by Aguilar-Toalá and co-workers [2], focused on the multifunctional analysis of chia seed (*Salvia hispanica L*.) bioactive peptides using peptidomics, molecular simulation techniques and ensemble molecular docking analysis. The third submission, by Tonolo and co-workers [3], deals with Nrf2-activating bioactive peptides, K-8-K and S-10-S, which were proved to exert anti-inflammatory effects by inhibiting the NF-κB pathway. The fourth manuscript by Cerrato and co-workers [4] used high-resolution mass spectrometry to characterize the short endogenous peptidome in dry-cured ham samples at different processing stages (14, 22, and 34 months) to help the understanding on how they may affect its sensory and functional properties. The fifth manuscript, authored by Heres and co-workers [5], reported the identification and quantitation of bioactive and taste-related dipeptides PA, GA, VG, EE, ES, DA, and DG in low-salt Spanish dry-cured ham through the use of triple quadrupole mass spectrometry. Such dipeptides were also confirmed as anti-inflammatory and potential cardiovascular protectors. Coscueta and co-workers [6] author the sixth manuscript, which focused on the screening of novel bioactive peptides from goat casein, using in vitro validation and new computational biology technologies to predict peptide sequences. The seventh paper, authored by Abioye and co-workers [7], reported the identification of three tetrapeptides, TNGQ, MANT, and YMSV, which inhibited islet amyloid polypeptide fibrillation. Their findings indicated the potential of TNGQ in the development of peptide-based anti-fibrillation and antidiabetic nutraceuticals. In the eighth manuscript, Santos and co-workers [8] reported a novel casein-derived antibacterial polypeptide produced through a cheese whey fermentation methodology and capable of inhibiting matrix metalloproteases MMP-2 and MMP-9. The ninth and last manuscript, submitted by Chen and co-workers [9], is focused on the proteolytic capacity of sea bass by-product fermented with lactic acid bacteria, followed by hydrolysis with Protease N under high hydrostatic pressure and the identification of the resulting peptides SAQ, PW and VGGT with hypocholesterolemic activity via the inhibition of cholesterol micelle formation.

The guest editors would like to thank the contributors, peer reviewers, and journal’s managers for their kind support and contribution to this issue, and also thank the MDPI´s publishing team for expediting its handling. The guest editors hope and wish that this issue will be of interest to the readers of the *International Journal of Molecular Sciences* and the scientific community.

## References

[1] Martineau-Côté D., Achouri A., Wanasundara J., Karboune S., L’Hocine L. (2022). Health Beneficial Bioactivities of Faba Bean Gastrointestinal (In Vitro) Digestate in Comparison to Soybean and Pea. Int. J. Mol. Sci..

[2] Aguilar-Toalá J.E., Vidal-Limon A., Liceaga A.M. (2022). Multifunctional Analysis of Chia Seed (Salvia hispanica L.) Bioactive Peptides Using Peptidomics and Molecular Dynamics Simulations Approaches. Int. J. Mol. Sci..

[3] Tonolo F., Folda A., Scalcon V., Marin O., Bindoli A., Rigobello M.P. (2022). Nrf2-Activating Bioactive Peptides Exert Anti-Inflammatory Activity through Inhibition of the NF-κB Pathway. Int. J. Mol. Sci..

[4] Cerrato A., Aita S.E., Capriotti A.L., Cavaliere C., Montone A.M.I., Montone C.M., Laganà A. (2022). Investigating the Short Peptidome Profile of Italian Dry-Cured Ham at Different Processing Times by High-Resolution Mass Spectrometry and Chemometrics. Int. J. Mol. Sci..

[5] Heres A., Gallego M., Mora L., Toldrá F. (2022). Identification and Quantitation of Bioactive and Taste-Related Dipeptides in Low-Salt Dry-Cured Ham. Int. J. Mol. Sci..

[6] Coscueta E.R., Batista P., Gomes J.E.G., da Silva R., Pintado M.M. (2022). Screening of Novel Bioactive Peptides from Goat Casein: *In Silico* to *In Vitro* Validation. Int. J. Mol. Sci..

[7] Abioye R.O., Okagu O.D., Udenigwe C.C. (2022). Disaggregation of Islet Amyloid Polypeptide Fibrils as a Potential Anti-Fibrillation Mechanism of Tetrapeptide TNGQ. Int. J. Mol. Sci..

[8] Santos M.I., Lima A., Mota J., Rebelo P., Ferreira R.B., Pedroso L., Ferreira M.A., Sousa I. (2021). Extended Cheese Whey Fermentation Produces a Novel Casein-Derived Antibacterial Polypeptide That Also Inhibits Gelatinases MMP-2 and MMP-9. Int. J. Mol. Sci..

[9] Chen G.W., Lin H.-T.V., Huang L.-W., Lin C.-H., Lin Y.-H. (2021). Purification and Identification of Cholesterol Micelle Formation Inhibitory Peptides of Hydrolysate from High Hydrostatic Pressure-Assisted Protease Hydrolysis of Fermented Seabass Byproduct. Int. J. Mol. Sci..

